# A tasty cultural event

**DOI:** 10.1007/s10194-012-0472-3

**Published:** 2012-07-22

**Authors:** Paolo Martelletti

**Affiliations:** Department of Clinical and Molecular Medicine, Sapienza University of Rome, Rome, Italy

## JHP rumps up

After the usual year of editorial silence, I come back to comment the Impact Factor (IF) attribution for the year 2011. 28 June 2012 has been an important day for every scientific journal, and the wish to share the taste of the results of an entire year of work is strong.

The IF, the more known bibliometric index, universally accepted for stating the quality of a journal, has been published on that day on the 2011 Journal Citation Reports^®^ (Thomson Reuters 2011), and data say that The Journal of Headache and Pain has seen a growth of the 21 % compared to the previous year. The absolute value of the IF of The Journal of Headache and Pain for the year 2011 is 2.427, a result of excellence, both *per se* and compared to the IF of the other journals working in the same area [1].

I publicly thank everyone who, in this occasion, has expressed appreciation on the obtained result. Nothing anyway would have been possible without the weighty management structure of Springer, the continuous and qualified contribution of the reviewers, the shared effort from the Editorial and the Advisory Board, creating the conditions for this extraordinary vehicle that is *The Journal of Headache and Pain* to deliver a great performance.

## Numbers taking flight once again

An analysis of the result is due, and some simple data (2011 vs. 2010) are self-descriptive:

- The distribution of the citations per year has increased of 43 %
- The ranking in the subject category Clinical Neurology is 81/191 (+10)
- The ranking in the subject category Neuroscience is 142/243 (+19)
- The percentage of self-citations has remained steady (21 %)

Additionally, more data related to 2011 versus 2010 should be reported:

- The total number of submissions has increased by 10 %
- The number of downloaded papers has increased by 62 %
- The rejection rate has increased by 18 %

More technical detailed analyses are released as Supplemental Material to this article, for those who wish to have a more in-depth information.

These data must be contextualized in a global economic recession which strongly penalizes biomedical research, and produced an overt slowdown in filling the pipeline of new drugs for headache.

## The shared backyard

The headache research has changing borders, and this peculiarity has always produced ideas and results of excellence. As for the headache health care services, now undeniably worthy if organized in a multi-disciplinary way, also the headache research can gain new lymph by looking to its next-door neighbours and by sharing with them its own backyard.

The notion that a multiple approach to chronic headache is a *viaticum* for the management success is by now ascertained. Togetherness, needless to say, has its clashes but carries a potential for synergies much greater than when different scientists remain in their own backyards. Genetics of common headache disorders, electrical neuromodulation, toxicology, rehabilitation, etc. are the borders where our research must continue to face its challenges tomorrow. The Journal of Headache and Pain will focus on all the innovative areas which, together with the historical ones, shape the forthcoming future of the research.

## Can-do-attitude

I am optimistic about the future of The Journal of Headache and Pain. The basis of the journal growth wasn’t politically determined. The fundamentals have revolved around an ecosystem of independency, efficiency, and innovation with appropriate levels of regulations.

The solid relationship with the affiliated societies, European Headache Federation and Lifting The Burden will be further strengthened, thus to complete the journal, which strongly aims to contribute to the birth of strategies which model the foundations of new ideas, instead of being a simple receptacle for them.

Furthermore, the choice to render the journal freely accessible as Open Access has proved to be successful [2, 3], and will surely give more tasty fruits next year, when we will meet again for our usual end-of-June appointment. I confide in accessing sponsorships which will allow us to extend this option, alternatively to the also modern “fee for Authors” choice. It is widely accepted by now that a research group who wants to make its data accessible to everyone foresees publication costs in the budget of a research protocol.

Finally, in this last year of my second 5-year-tenure as Editor-in-Chief, the journal will keep its can-do-attitude operative, accepting innovative ideas for promoting a most wide-ranging and inter-cultural headache research.

## Electronic supplementary material

Below is the link to the electronic supplementary material.

Supplementary material 1 (DOC 34 kb)
