# Supplementary material for: A tasty cultural event
Source: J Headache Pain. 2012 Jul 22;13(6):435–6. doi: 10.1007/s10194-012-0472-3 (PMC3464471; doi:10.1007/s10194-012-0472-3)
Supplement: Supplementary file 1 — Supplementary material 1 (DOC 34 kb) [file 10194_2012_472_MOESM1_ESM.doc]

**Electronic Supplementary Material**

| ***The Journal of Headache and Pain – IF 2011*** | | | | |
| --- | --- | --- | --- | --- |
| IF | Immediacy Index | Cited Half-life | Total Cites | Eigenfactor™ Score |
| 2.427 | 0.941 | 3.2 | 928 | 0.00277 |
| **Subject Category *Neurosciences*** | | | | |
| Median IF | Aggregate Immediacy Index | Aggregated Cited-Half-Life | JHP Ranking | Quartile |
| 2.759 | 0.782 | 7.4 | 142/243 | Q3 |
| **Subject Category *Clinical Neurology*** | | | | |
| Median IF | Aggregate Immediacy Index | Aggregated Cited-Half-Life | JHP Ranking | Quartile |
| 2.119 | 0.608 | 7.1 | 81/191 | Q2 |
|  | | | | |
| **ΔIF JHP vs Median IF Neurosciences = -0.332 and vs Median IF Clinical Neurology = +0.308** | | | | |
| Source: Journal Citation Reports® (Thomson Reuters, 2011)  JCR Science Edition 2011 : <http://admin-apps.isiknowledge.com/JCR/JCR>  Released, June 28^th^, 2012, 7:00 PM, GMT | | | | |
